# Molecular identification of *Sarcocystis halieti* in the muscles of two species of birds of prey from Spain

**DOI:** 10.1186/s13071-021-04921-0

**Published:** 2021-08-18

**Authors:** Petras Prakas, Antonio Bea, Evelina Juozaitytė-Ngugu, Iñaki Olano, Diego Villanúa, Saulius Švažas, Dalius Butkauskas

**Affiliations:** 1 Nature Research Centre, Akademijos 2, 08412 Vilnius, Lithuania; 2 Ekos Estudios Ambientales S.L.U., Donostia Etorbidea 2, Bajo 2, 20160 Lasarte, Spain; 3 Navarra Environmental Management GAN-NIK, Calle Padre Adoain 219, 31015 Pamplona, Spain

**Keywords:** *Sarcocystis halieti*, Birds of prey, Molecular identification, ITS1, 28S rRNA

## Abstract

**Background:**

Members of the genus *Sarcocystis* are protozoan parasites characterized by a prey–predator two-host life-cycle. Sarcocysts are formed in the muscles or central nervous system of the intermediate host (IH), while sporocysts develop in the small intestine of the definitive host (DH). Various birds of prey have been confirmed to be DH for *Sarcocystis* spp. Three *Sarcocystis* species, *S*. *wobeseri*, *S*. *halieti* and *S*. *falcatula*, have been identified in the muscles of birds of prey, of which the latter are known to be pathogenic and can cause encephalitis in various birds. The aim of this study was to identify *Sarcocystis* spp. in the muscles of birds of prey from Spain.

**Methods:**

Between 2019 and 2020, muscle tissue samples taken from 59 birds of prey admitted to the Wildlife Recovery Centre in Ilundain (Navarra, Spain) were examined for the presence of *Sarcocystis* spp. Sarcocysts in fresh squashed samples were morphologically characterized under the light microscope (LM). *Sarcocystis* spp. were identified by means of 28S ribosomal RNA and internal transcribed spacer 1 sequence analysis.

**Results:**

Microscopic examination of squashed tissue samples stained with methylene blue revealed the presence of sarcocysts in three of the 59 (5.1%) birds examined. Only one sarcocyst type was observed under the LM. Sarcocysts were thread-like (1050–2160 × 130–158 μm) and had a thin (0.7–1.4 μm) and smooth cyst wall. Septa divided the cysts into compartments filled with banana-shaped (5.9 × 1.7 μm) bradyzoites. On the basis of DNA sequence results, *S*. *halieti* was identified in the western marsh harrier (*Circus aeruginosus*) and the black kite (*Milvus migrans*) for the first time. Sarcocysts of *S*. *halieti* were shorter and wider compared to those observed in the great cormorant (*Phalacrocorax carbo*) and the herring gull (*Larus argentatus*). According to current knowledge, *S*. *halieti* may infect birds belonging to four different orders: Suliformes, Charadriiformes, Strigiformes and Accipitriformes.

**Conclusions:**

This is the first report of *S*. *halieti* in the western marsh harrier and the black kite as IH. So far, little research has been conducted on birds of prey as IH for *Sarcocystis* spp. These results indicate that further studies combining morphological, histopathological, and molecular methods are required.

**Graphical abstract:**

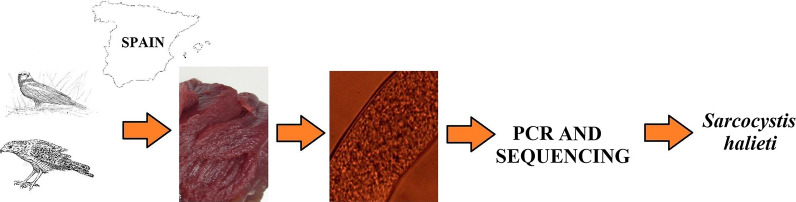

## Background

Members of the genus* Sarcocystis* are protozoan parasites characterized by an obligatory two-host prey–predator life-cycle. Asexual multiplication with sarcocyst formation occurs in the muscles and/or central nervous system (CNS) of the intermediate host (IH), whereas sexual multiplication stages, oocysts/sporocysts develop in the small intestine of the definitive host (DH) [1]. Currently, 27 valid *Sarcocystis* spp. using birds as IH are known [2].

Raptors are indicators of biodiversity and environmental health, and are are recognized in ecosystems as top predators and scavengers, and as flagship species [3]. Various birds of prey (eagles, hawks, falcons and owls) have been examined as possible DH [1]. A number of investigations have also looked at birds of prey as IH of *Sarcocystis* spp. [4–11]. The pathogenic species *Sarcocystis* *falcatula* may cause encephalitis in the free-ranging great horned owl (*Bubo virginianus*) [6], the golden eagle (*Aquila chrysaetos*) and the bald eagle (*Haliaeetus leucocephalus*) [7]. Likewise, an undescribed *Sarcocystis* sp. causing encephalitis has been detected in an immature northern goshawk (*Accipiter gentilis atricapillus*) from Minnesota [4]. Recently, *Sarcocystis* *wobeseri* was identified in the pectoral and cardiac muscles of the white-tailed sea eagle (*Haliaeetus albicilla*) [10]. Also, *Sarcocystis* *halieti* was detected in the brain and muscle tissue of a juvenile free-ranging little owl (*Athene noctua*) [11]. Thus, to date sarcocysts of three *Sarcocystis* spp., *S*. *falcatula*, *S*. *halieti* and *S*. *wobeseri*, have been recorded in the brains and tissues of birds of prey [6, 7, 10, 11]. In another study, three morphological types of sarcocysts were detected in the Eurasian buzzard (*Buteo buteo*) and the long-eared owl (*Asio otus*), with one of the sarcocysts in the owl identified as *S*. *otus* [12]. However, this latter species is considered to be invalid [1].

In this article, we describe the molecular identification of *S. halieti* in the muscles of birds of prey from Spain.

## Methods

Between 2019 and 2020 tissue samples of the leg muscles of 59 birds of prey (Accipitriformes, Falconiformes and Strigiformes) were examined for *Sarcocystis* spp. (Table 1). The birds had been admitted to the Wildlife Recovery Centre in Ilundain (Navarra) (Spain). The samples were taken by the Center’s veterinary staff while carrying out their routine diagnostic protocol for the cause of death of the birds, which were either brought to the Center as dead specimens or died there. This center is under the jurisdiction of the Government of Navarra and is managed by a public company, GAN-NIK. Muscle samples were kept frozen (− 20 °C) until studied for morphological detection of the sarcocysts. The prevalence of sarcocysts and infection intensity were evaluated in methylene blue-stained muscle samples, as previously described [13].

**Table 1:** Birds of prey (*n* = 59) from Navarra (Spain) examined for *Sarcocystis* spp.

Order	Common name (species)	Infected/examined
Accipitriformes	Black kite (*Milvus migrans*)	2/6
Accipitriformes	Western marsh harrier (*Circus aeruginosus*)	1/1
Accipitriformes	Booted eagle (*Hieraaetus pennatus*)	0/2
Accipitriformes	Common buzzard (*Buteo buteo*)	0/4
Accipitriformes	Eurasian sparrowhawk (*Accipiter nisus*)	0/1
Accipitriformes	Red kite (*Milvus milvus*)	0/9
Accipitriformes	European honey buzzard (*Pernis apivorus*)	0/1
Accipitriformes	Northern goshawk (*Accipiter gentilis*)	0/3
Accipitriformes	Griffon vulture (*Gyps fulvus*)	0/4
Falconiformes	Common kestrel (*Falco tinnunculus*)	0/7
Strigiformes	Eurasian scops owl (*Otus scops*)	0/13
Strigiformes	Long-eared owl (*Asio otus*)	0/2
Strigiformes	Brown owl (*Strix aluco*)	0/3
Strigiformes	Little owl (*Athene noctua*)	0/3

Muscle samples of infected birds were delivered to the Laboratory of Molecular Ecology, Nature Research Centre, Vilnius, Lithuania for detailed morphological and molecular analysis. The morphological characterization of sarcocysts and bradyzoites was performed in fresh-squashed samples. Sarcocysts with a small amount of host tissue were excised using two preparation needles, transferred to a drop of water on a microscope slide and measured under a light microscope (LM) at ×40–×1000 magnification.

Genomic DNA was isolated from individual sarcocysts using the GeneJET Genomic DNA Purification Kit (Thermo Fisher Scientific Baltics, Vilnius, Lithuania). Partial 28S rDNA was amplified using the KL-P1F/KL-P2R primer pair [14], and the complete internal transcribed spacer 1 (ITS1) region was amplified using the SU1F/5.8SR2 primer pair [15]. The thermocycling conditions of the PCRs were as described previously [13]. Visualization, purification, and sequencing of PCR products were carried out using a previously described protocol [16]. The sequences obtained in this study were compared with those of various *Sarcocystis* spp. using the nucleotide BLAST program (megablast option) [17]. The multiple alignment was conducted using the MUSCLE algorithm loaded in MEGA7 software [18]. Selection of a nucleotide substitution model and phylogenetic analysis under Bayesian inference were carried out using TOPALi v2.5 [19].

Sarcocysts were detected in methylene-blue stained muscle samples from the leg muscles of one of the black kites (*Milvus migrans*); however, they were not observed in the freshly squashed samples not stained with methylene blue. Therefore, the muscle sample of this bird was digested with pepsin according to the modified protocol of Dubey et al. [1]. Specifically, 5 g of leg muscle tissue was cut into small pieces and suspended in 15 ml of saline solution (0.9%). The suspension was then homogenized in a commercial blender at top speed for 2 min with breaks. The homogenate was transferred into a 150-ml flask and 15 ml of digestion solution was added (pepsin, 0.26 g; NaCl 0.5 g; water up to 15 ml; and 37% HCl to pH 1.1). The entire contents of the flask were incubated at 37 °C for 2 h and the suspension was used for DNA extraction. Genomic DNA was extracted as described above. External PCR primers were SU1F/5.8SR2 [15], and internal primers GsShalF1 (5′-GATAATTGACTTTACGCGCCATTAC-3′) and GsShalR1 (5′GTGCACATCCATATATGCTCATTCT-3′) were designed specifically for this study. The first run of a nested PCR assay was conducted as described in [13]. The second run of a nested PCR assay was carried out in a final volume of 12.5 μl consisting of 6.3 μl of DreamTaq PCR Master Mix (Thermo Fisher Scientific Baltics), 0.5 μM of each primer, 1 μl from the first run of PCR and nuclease-free water. The thermocycling conditions were: 1 cycle at 95 °C, 5 min; then 94 °C/45 s, 65 °C/45 s, 72 °C/50 s for 35 cycles; with a final extension at 72 °C, 7 min. Visualization, purification and sequencing of PCR products were performed as described above. Sequences generated in the present study were deposited in GenBank under accession numbers MW926916–MW926917 and MW929599–MW929601.

## Results

Sarcocysts were detected in tissue samples stained with methylene blue examined under the LM in three of the 59 (5.1%) birds of prey: one western marsh harrier (*Circus aeruginosus*; isolate CaEs1) and two black kites (*Milvus migrans*; isolates MmEs1 and MmEs2). Two sarcocysts (isolates CaEs1 and MmEs1) were excised from fresh samples and subjected to amplification and sequencing of 28S rRNA and ITS1. Sarcocysts were not found in a fresh muscle sample from a single black kite (isolate MmEs2); therefore, this sample was digested and *Sarcocystis* DNA was amplified using a nested PCR targeting the ITS1 region.

**Fig. 1:**
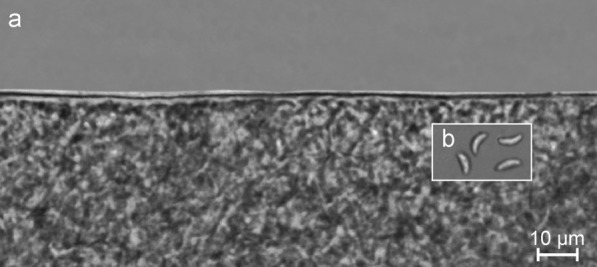
Morphology of *Sarcocystis halieti* in tissue sample taken from the leg muscle of the black kite (*Milvus migrans*), fresh preparation. **a** Fragment of the smooth cyst wall, **b** banana-shaped bradyzoites

From three to seven sarcocysts were observed in 1 g of methylene blue-stained muscle sample. Under the LM, one type of sarcocyst was observed. Sarcocysts seemed to be thread-like, 1560 × 143 μm (range: 1050–2160 × 130–158 μm; *n* = 6) in size, with a thin (0.7–1.4 μm), apparently smooth cyst wall (Fig. 1a). The sarcocysts were clearly divided by septa into compartments filled with mature banana-shaped bradyzoites, 5.9 × 1.7 μm (range: 4.8–7.1× 1.3–2.1 μm; *n* = 45) in size (Fig. 1b).

**Fig. 2:**
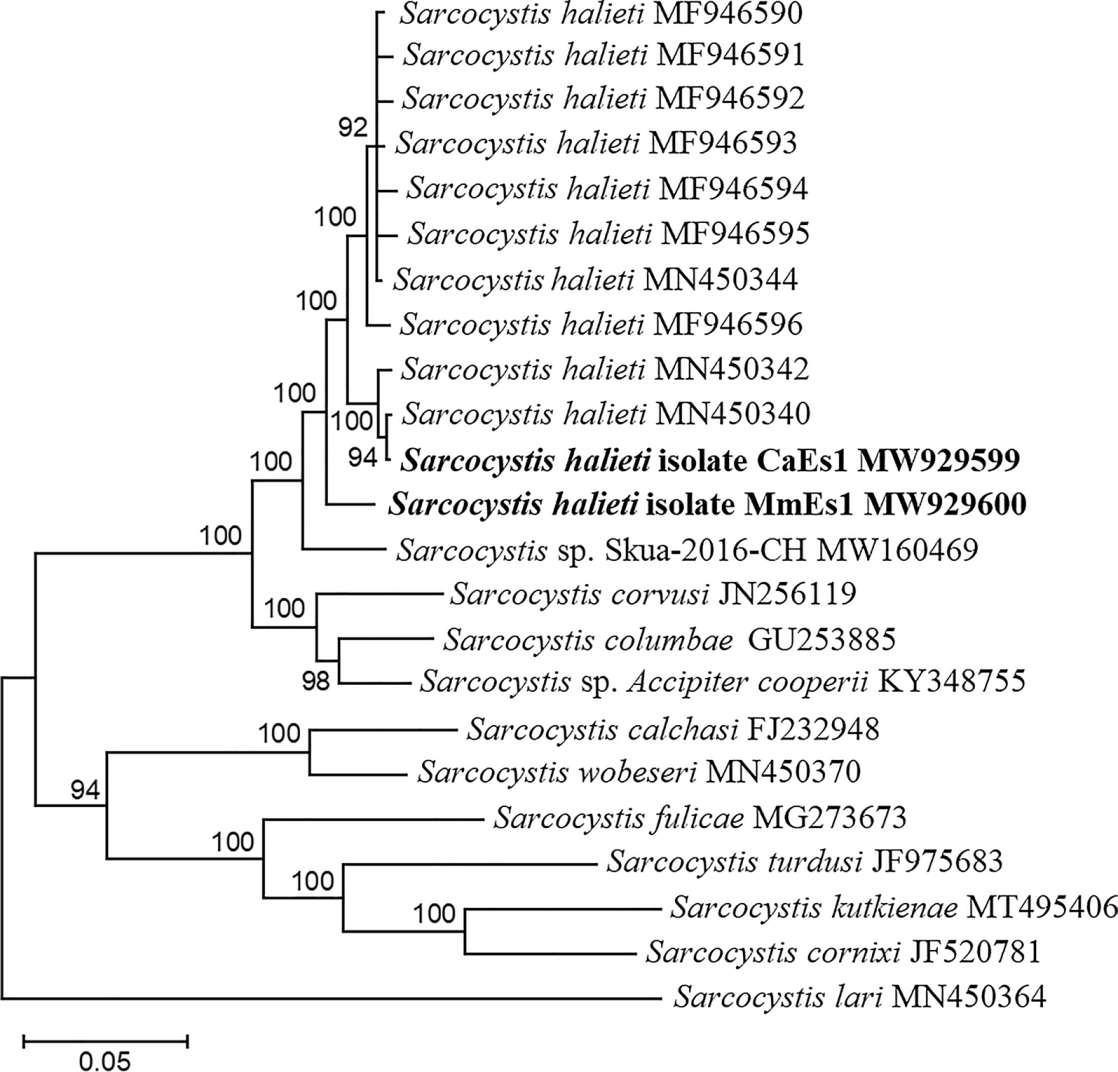
Phylogenetic tree of selected *Sarcocystis* spp. based on internal transcribed spacer (ITS1) sequences. The tree was constructed using Bayesian methods, scaled according to the branch length and rooted on *Sarcocystis lari*. The final alignment of the ITS1 sequences contained 23 taxa and 982 aligned nucleotide positions. Numbers next to branches show the posterior probability support values. Sequences generated in the present study are indicated in boldface

The obtained 830-bp ITS1 sequence from the western marsh harrier (*Circus aeruginosus*) (isolate: CaEs1) was 100% identical with *S*. *halieti* from the great cormorant (*Phalacrocorax carbo*) (JQ733513, MH130209) and from the herring gull (*Larus argentatus*) (MN450340, MN450341). The 569-bp ITS1 sequence generated from the digested muscle sample of the black kite (MmEs2) demonstrated 100% identity with *S*. *halieti* from the herring gull (MN450344–MN450356) and from the white-tailed eagle (MF946589, MF946590), whereas the 830-bp ITS1 sequences obtained for sarcocyst isolated from the black kite (MmEs1) showed 97.2–97.7% similarity to *S*. *halieti* from the herring gull (MN450340–MN450356), the white-tailed eagle (MF946589–MF946596) and the great cormorant (JQ733513, MH130209), had 96.0% similarity to *Sarcocystis* sp. from the Chilean skua (*Stercorarius chilensis*) (MW160469), 93.2% similarity to *Sarcocystis* sp. from the Cooper’s hawk (*Accipiter cooperii*) (KY348755) and 92.3–92.5% similarity to *S*. *columbae* from the herring gull (MN450338, MN450339) and from the woodpigeon (*Columba palumbus*) (GU253885, HM125052). The ITS1 sequences obtained from two black kites (MmEs1 and MmEs2) displayed 97.0% similarity. In the ITS1 phylogenetic tree, the sequences from the black kites and the western marsh harrier were placed in one cluster together with those from *S*. *halieti* and *Sarcocystis* sp. from the Chilean skua (Fig. 2). It should be noted that the sequence from the black kite (MmEs1) formed a sister branch to the other *S*. *halieti* sequences. The 1488-bp 28S rRNA sequence from the black kite (MmEs1) differed by one to two single nucleotide polymorphisms (SNPs) from those of *S*. *halieti* (JQ733512, MF946610, MH130210) and by seven SNPs from those of *S*. *columbae* (HM125053), while the 1508-bp 28S rRNA sequence from the western marsh harrier (CaEs1) demonstrated 99.3–100% identity with that from *S*. *halieti*. Thus, on the basis of the molecular examination, *S*. *halieti* was identified in two black kites and a single western marsh harrier.

## Discussion

The results of the present study reveal new IH for *S*. *halieti*, which we identified in the black kite and the western marsh harrier for the first time. A juvenile little owl was recently diagnosed with granulomatous encephalitis and muscular sarcocysts caused by *S*. *halieti* [11]; earlier studies had detected *S*. *halieti* in the great cormorant [20] and the herring gull [21]. Our results extend the body of knowledge on *S*. *halieti* specificity for the IH and indicate that this species is able to form sarcocysts in birds belonging to at least four different orders: Accipitriformes (present study), Charadriiformes [21], Strigiformes [11] and Suliformes [20]. Other avian *Sarcocystis* spp. (i.e. *S*. *calchasi*, *S*. *columbae*, *S*. *falcatula* and *S*. *wobeseri*) can also form sarcocysts in IH belonging to different orders [1, 10, 21, 22]. The development of molecular methods and expansion of the diversity of the host species examined had led to the detection of the known *Sarcocystis* spp. in different bird orders [22]. Such investigations are of particular importance in terms of pathogenic species. It should be noted that highly pathogenic *Sarcocystis* spp., such as *S*. *neurona*, *S*. *canis*, *S*. *felis*, *S*. *calchasi* and *S*. *falcatula*, are multi-host adapted [1], as exemplified by the recent detection of *S*. *halieti*-associated encephalitis in a little owl from Germany [11]. Therefore, further investigation of the pathogenesis of *S*. *halieti* in various birds is required and extensive histopathological studies of this pathogenic species are recommended.

Sarcocysts of *S*. *halieti* detected in the muscles of birds of prey seemingly differed morphologically from those previously described in other IH. For comparative purposes, sarcocysts of *S*. *halieti* isolated from the leg and neck muscles of the great cormorant were very long, up to 6.5 × 0.1 mm [20], whereas sarcocysts excreted from the leg muscles of the herring gull were from 3960 μm to 7930 μm in length and from 43 μm to 128 μm in width [21]. Sarcocysts identified in the leg muscles of black kites and the western marsh harrier were, in comparison, shorter and wider (1050–2160 × 130–158 μm). *Sarcocystis halieti* sarcocysts of various shapes may be associated with diverse types of host anatomical structure. In terms of the distribution of muscle forces, accipitrids, falconids and strigiforms tend to possess greater proportions of distally inserted digital flexor musculature (53–64%, on average) [23]. On the other hand, the size of sarcocysts might depend on the contraction of the muscle fibers and the amount of pressure applied to the cover slip [1].

In the present study, sarcocysts were observed in three of the 59 (5.1%) birds of prey from Spain that were studied, with the help of methylene blue staining. A similar low prevalence of infection was detected in raptorial birds from Germany (3/79, 3.8%) [12] and Australia (5/38, 13.2%) [24] by histological examination. Based on pepsin digestion, significantly higher infection rates (52/114, 45.6%) were reported in birds of prey from the USA [5]. Whereas when examined by an immunofluorescence antibody test, eight of 72 (11.1%) raptorial birds from Brazil were found to be positive for *Sarcocystis* spp. [25]. Altogether, previous studies suggest that the prevalence of *Sarcocystis* spp. infection in raptorial birds varies depending on the method used for examination of the tissue samples.

Due to a lack of published data from comprehensive microscopic examinations, it is difficult to compare the morphology of the sarcocysts of *S*. *halieti* identified in the present study with those observed in other birds of prey in previous studies. Based on LM examination, two types of sarcocysts were reported in bald eagles from the USA [26]. The first type of sarcocyst was microscopic, had a thin cyst wall with spines and contained bradyzoites 5 × 1 µm in size; the second type (type II) was immature and had a 2-µm-thick striated cyst wall [26]. These sarcocysts are not similar to those observed in our study. However, type II sarcocysts were detected in the Eurasian buzzard [12]; these measured 694–1850 × 42–235 µm, had a seemingly smooth cyst wall and resembled those of *S*. *halieti*. A histological study detected thin-walled (0.5 µm) sarcocysts with a smooth surface and no visible protrusions in the cardiac muscle of the white-tailed sea eagle from Norway [27]; the length of the sarcocysts was not determined, but the diameter of the largest cyst measured was 40 µm. Subsequently, *S*. *wobeseri* was identified in the muscles of the white-tailed sea eagle from the UK [10]. Based on current knowledge, the sarcocysts of *S*. *halieti* and *S*. *wobeseri* are morphologically indistinguishable [21]. Lastly, thin-walled (≤ 1 μm) and thick-walled (2–4 μm) sarcocysts were detected in the muscles of raptors from the south-eastern USA [8]; the thin-walled sarcocysts might represent those of *S*. *halieti*, *S*. *wobeseri* or of an otherwise not yet confirmed *Sarcocystis* spp.

The most detailed morphological examination of sarcocyst structure occurs using transmission electron microscopy (TEM) [1]. To date, only a few studies have used TEM to describe sarcocysts in raptorial birds [7, 12]. Based on TEM, the sarcocyst of *Sarcocystis* sp. found in the Eurasian buzzard and having a thin (up to 1.2 µm) and wavy cyst wall [12] is similar to that of *S*. *halieti* [20]. However, sarcocysts of *S*. *halieti* under the transmission electron microscope are very similar to those of *S*. *calchasi*, *S*. *corvusi*, *S*. *fulicae*, *S*. *columbae*, *S*. *lari* and *S*. *wobeseri* [1, 20, 28]. Also, four *Sarcocystis* species (*S*. *columbae*, *S*. *halieti*, *S*. *lari* and *S*. *wobeseri*) were identified in the herring gull [21]. Hence, molecular methods are needed for decisive discrimination of *Sarcocystis* spp. using birds as IH.

The most recent studies on *Sarcocystis* spp. in birds of prey have focused on the diagnosis of this apicomplexan genus using muscle digestion and subsequent nested PCR [9] or an immunofluorescence antibody test [25]. These methods are relatively sensitive to the detection of *Sarcocystis* spp. However, even with these methods, morphological characteristics of sarcocysts, which are important to achieve phenotypic diagnosis of *Sarcocystis* spp., cannot be determined. In summary, studies on the role of birds of prey as IH of *Sarcocystis* spp. are fragmentary, and it is difficult to compare the results obtained by different morphological and molecular methods.

Eight *Sarcocystis* spp., namely *S*. *accipitris*, *S*. *alectoributeonis*, *S*. *calchasi*, *S*. *columbae*, *S*. *cornixi*, *S*. *halieti*, *S*. *lari* and *S*. *turdusi*, using birds as IH are transmissible by birds of prey [1, 27, 29]. The white-tailed sea eagle and the Eurasian sparrow hawk (*Accipiter nisus*) (family Accipitridae) have been confirmed to be DH of *S*. *halieti* [27, 29]. In the present study, two bird species also belonging to family Accipitridae were shown to be IH of *S*. *halieti*. Hence, there is a possibility that accipitrids might be infected with *S*. *halieti* through cannibalism. Such diet behavior is common among raptorial birds, especially among members of the Accipitridae family (65.5%) [30]. Future studies on the transmission of *S*. *halieti* are needed.

## Conclusions

In the present study, *S*. *halieti* was identified in the black kite and the western marsh harrier from Navarra (Spain) by means of 28S rDNA and ITS1 sequence analysis. This is the third *Sarcocystis* species to be detected in the muscles of birds of prey. Studies on *Sarcocystis* spp. from birds of prey are fragmentary. Therefore, further combined morphological, histopathological and molecular methods should be employed to provide a comprehensive description of *Sarcocystis* spp. found in birds of prey.

## Data Availability

Data supporting the conclusions of this article are included within the article. The 28S rRNA and ITS1 sequences generated in the present study were submitted to the GenBank database under accession numbers MW926916–MW926917 and MW929599–MW929601, respectively.

## Abbreviations

CNS: Central nervous system
DH: Definitive host
IH: Intermediate host
ITS1: Internal transcribed spacer 1
LM: Light microscope
rRNA: Ribosomal RNA
SNP: Single nucleotide polymorphisms
TEM: Transmission electron microscopy
